# Grain Boundary Mechanisms and Regulation in the Hydriding Corrosion of Metallic Uranium

**DOI:** 10.3390/ma19214609

**Published:** 2026-10-30

**Authors:** Tianyu Zhang, Longxian Li, Min Zhu, Yan Li, Longfei Pu, Chengxuan Peng

**Affiliations:** Naval University of Engineering, Wuhan 430030, China; m25182709@nue.edu.cn (T.Z.);

**Keywords:** metallic uranium, hydriding corrosion, grain boundary, hydrogen segregation, interfacial regulation

## Abstract

Metallic uranium has important applications in nuclear fuels, nuclear materials, and related engineering fields because of its distinctive nuclear physical properties. However, its high chemical reactivity makes it susceptible to hydriding corrosion in hydrogen-containing environments. The substantial volumetric expansion associated with hydride formation, together with the resulting cracking and pulverization, severely compromises structural integrity and poses significant risks to long-term storage and safe service. Focusing on the role of grain boundaries in the hydriding corrosion of metallic uranium, this review summarizes current understanding of hydrogen ingress through the surface oxide layer, hydrogen segregation and migration at grain boundaries, and the regulation of these processes by elemental segregation. Defects and grain boundaries within the oxide layer can provide preferential pathways for hydrogen transport. In the metallic matrix, local structural distortion, excess free volume, and characteristic electronic structures at grain boundaries promote hydrogen trapping and accumulation, potentially facilitating hydride nucleation and intergranular damage by weakening interfacial cohesion. Metallic solutes affect the hydriding process primarily by modifying phase stability, hydrogen accumulation, and grain boundary cohesion, whereas the effects of non-metallic impurities depend on their states of occurrence, segregation tendencies, and co-segregation interactions with hydrogen. Grain boundary structure and local chemistry therefore jointly govern hydrogen ingress, accumulation, and damage evolution, making them central to understanding and regulating localized hydriding corrosion in metallic uranium.

## 1. Introduction

Uranium materials are susceptible to corrosion under service conditions, with hydriding corrosion being one of the primary degradation mechanisms that threaten their structural integrity and service life [1]. This process leads to surface pulverization and a progressive loss of mechanical integrity, thereby compromising long-term storage, service stability, and operational reliability. Therefore, elucidating the fundamental mechanisms of hydriding corrosion and developing effective mitigation strategies are essential for ensuring the safe utilization of metallic uranium and for supporting its long-term storage.

Hydriding corrosion of metallic uranium refers to a sequence of physicochemical processes that occur during service or storage when uranium interacts with hydrogen in the surrounding environment. These processes involve surface adsorption, molecular dissociation, atomic diffusion, and subsequent chemical reactions, ultimately leading to the formation of uranium hydride and the associated loss of structural integrity and material performance. Fundamentally, hydriding corrosion is a coupled process involving interfacial uranium–hydrogen interactions and the dissolution and diffusion of hydrogen within the metallic matrix. Extensive experimental observations have shown that hydriding corrosion in uranium does not proceed uniformly but instead exhibits highly localized pitting behavior, with corrosion pits preferentially nucleating and rapidly propagating at specific microstructural sites. Notably, the majority of these sites are located at grain boundaries, indicating a pronounced tendency for grain-boundary-assisted nucleation. This behavior suggests that grain boundaries, as structurally and chemically heterogeneous regions within the microstructure, play a critical role in hydrogen transport, accumulation, and the subsequent corrosion reaction. Elucidating the interactions between grain boundaries and hydrogen is therefore essential for understanding the mechanisms of hydriding corrosion in metallic uranium and constitutes a central focus of this review.

As ubiquitous planar defects in polycrystalline materials, grain boundaries exert a profound influence on macroscopic properties through their distinct structural, chemical, and mechanical characteristics. In metallic uranium, grain boundaries not only serve as preferential sites for hydrogen segregation, but their intrinsic stability also directly affects the resistance of the material to corrosion and mechanical failure. In engineering uranium alloys, selected metallic alloying elements are commonly introduced to improve properties such as phase stability and mechanical strength. The segregation of these solute atoms to grain boundaries can substantially modify the local electronic structure, grain boundary cohesion, and diffusion characteristics, thereby exerting complex effects on hydrogen trapping and chemical reactivity. In addition, non-metallic impurities that are unavoidably introduced during material processing and fabrication may also preferentially accumulate at grain boundaries, further altering grain boundary properties and their interactions with hydrogen.

A hallmark of hydriding corrosion in metallic uranium is the preferential initiation of localized corrosion along grain boundaries, indicating that these interfaces serve as favorable pathways for hydrogen transport and hydriding reactions. Motivated by this phenomenon, this review systematically summarizes recent advances in understanding hydrogen behavior at grain boundaries in uranium, covering the complete hydrogen transport pathway from grain boundaries in the surface oxide layer to those within the metallic substrate. Particular emphasis is placed on the adsorption, diffusion, and accumulation of hydrogen in different grain boundary environments, as well as the mechanisms by which grain boundary structure and local chemical environment influence hydrogen behavior. In addition, recent progress in regulating grain boundary susceptibility to hydriding through alloying elements and non-metallic impurities is reviewed. Finally, the key scientific challenges and outstanding issues in the current understanding of hydriding corrosion are discussed, with the aim of providing theoretical guidance for improving the reliability and long-term performance of uranium materials under complex service conditions. From the perspective of the nuclear fuel cycle, the long-term behavior of metallic uranium under storage and disposal conditions is a central concern for nuclear materials safety. In these scenarios, hydrogen arises from surface reaction with residual moisture or hydrogen-bearing gases. The localized hydriding corrosion addressed here is therefore directly relevant to the integrity of uranium fuel elements, the safe storage of uranium materials, and their long-term geological disposal. It should be noted that the present review focuses on the hydriding corrosion of unirradiated metallic uranium exposed to external hydrogen sources (hydrogen-bearing gases or moisture). For spent or irradiated fuel, hydrogen can additionally be generated internally through radiolysis and ternary fission; this scenario involves substantially different physics and is beyond the scope of the present review. Accordingly, this review focuses on the role of grain boundaries in the hydriding corrosion of metallic uranium, tracing hydrogen transport from the surface oxide layer to the metallic grain boundaries and examining how alloying elements and non-metallic impurities regulate this process.

## 2. Hydriding Corrosion of Metallic Uranium

### 2.1. Characteristics and Consequences of Hydriding Corrosion in Metallic Uranium

**Figure 1:**
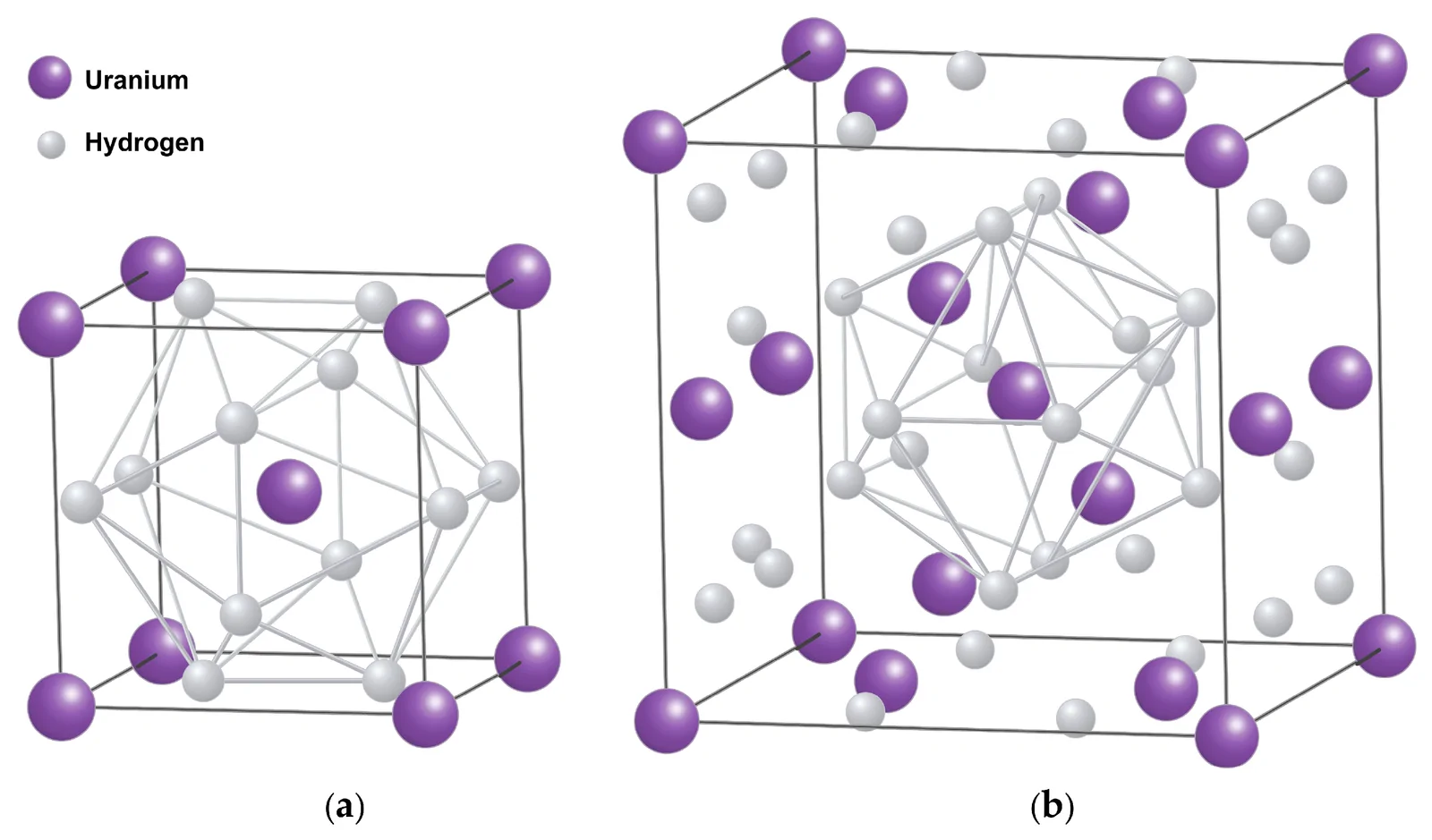
Crystal structures of uranium hydride: (**a**) α-UH_3_ (cubic) and (**b**) β-UH_3_, which possesses a larger unit-cell parameter. Uranium atoms are shown in purple and hydrogen atoms in grey. α-UH_3_ forms at relatively low temperatures, whereas β-UH_3_ becomes the predominant product at higher temperatures.

Owing to its exceptionally high chemical reactivity, metallic uranium exhibits a strong susceptibility to corrosion in a wide range of environmental media. Among the various degradation processes, hydriding corrosion arising from uranium–hydrogen interactions represents a critical threat to structural integrity and operational safety. The associated risks are further amplified by the radioactivity and toxicity of uranium, making hydriding corrosion a major safety concern in the storage and application of uranium-containing nuclear materials. At a given hydrogen partial pressure, the interaction between hydrogen gas and metallic uranium involves a sequence of processes, including physicochemical adsorption, dissociation of hydrogen molecules, diffusion of atomic hydrogen into the bulk, and hydrogen accumulation and nucleation at microstructural defects [2,3]. These processes ultimately lead to the formation of uranium hydride (UH_3_) at or near the material surface. Both the reaction pathway and the resulting hydride microstructure are strongly dependent on temperature and pressure. At temperatures below approximately 100 °C (i.e., near room temperature), the reaction produces α-UH_3_ with a cubic crystal structure, whereas β-UH_3_, which has a larger unit-cell parameter, becomes the predominant product above approximately 250 °C, as illustrated in Figure 1.

In practical service, hydrogen ingress leading to hydriding may arise through exposure to hydrogen-bearing gases or residual moisture during storage, or through the reaction of uranium with water, which liberates hydrogen that is re-absorbed by the metal. The underlying reaction is a redox process: hydrogen is reduced to hydride ions (H^−^), while uranium is oxidized from the metallic state (U^0^) to U^3+^ in UH_3_. The surface redox state is therefore central to the mechanism—the surface oxide, in which uranium adopts U^4+^ (possibly higher in hyper-stoichiometric UO_2+x_), acts as both a kinetic barrier to hydrogen ingress and the reactive surface for H_2_ dissociation, thereby determining where and how readily hydriding initiates.

The hazards associated with hydriding corrosion of metallic uranium arise not only from hydride formation itself, but also from the pronounced volume expansion and high chemical reactivity of the resulting hydride products. Macroscopically, hydriding corrosion is typically manifested as non-uniformly distributed pits on the material surface. Because UH_3_ has a substantially lower density than metallic uranium, its formation can produce a volume expansion of approximately 70%, generating considerable internal stresses that promote lattice distortion, interfacial cracking, and microcrack propagation, thereby severely degrading the mechanical and structural integrity of the material. Powdered UH_3_ is also highly reactive and can oxidize rapidly upon exposure to air or humid environments, releasing substantial heat and creating risks of spontaneous ignition or even dust explosion [4]. Such reactions may additionally generate radioactive aerosols, posing serious hazards to personnel and the environment. Moreover, hydrogen produced by the reaction between metallic uranium and water may be absorbed by the material and further promote hydriding, leading to a progressively sustained corrosion process. Continued pit growth ultimately disrupts the surface protective layer and geometric integrity of the material, providing pathways for corrosion to penetrate deeper into the substrate.

It should be noted that, although a general framework for the uranium–hydrogen reaction has been established, the high complexity of material microstructural features (such as defect distributions and oxide layer conditions) and environmental parameters (including temperature, pressure, and impurities) means that no unified and precise understanding has yet been reached regarding the exact mechanisms governing the incubation period, the critical conditions for nucleation, and the transition kinetics between various reaction stages. Deepening the understanding of these fundamental processes at a mechanistic level is essential for the accurate prediction and effective control of hydriding corrosion in metallic uranium.

### 2.2. Fundamental Characteristics of Grain Boundaries

**Figure 2:**
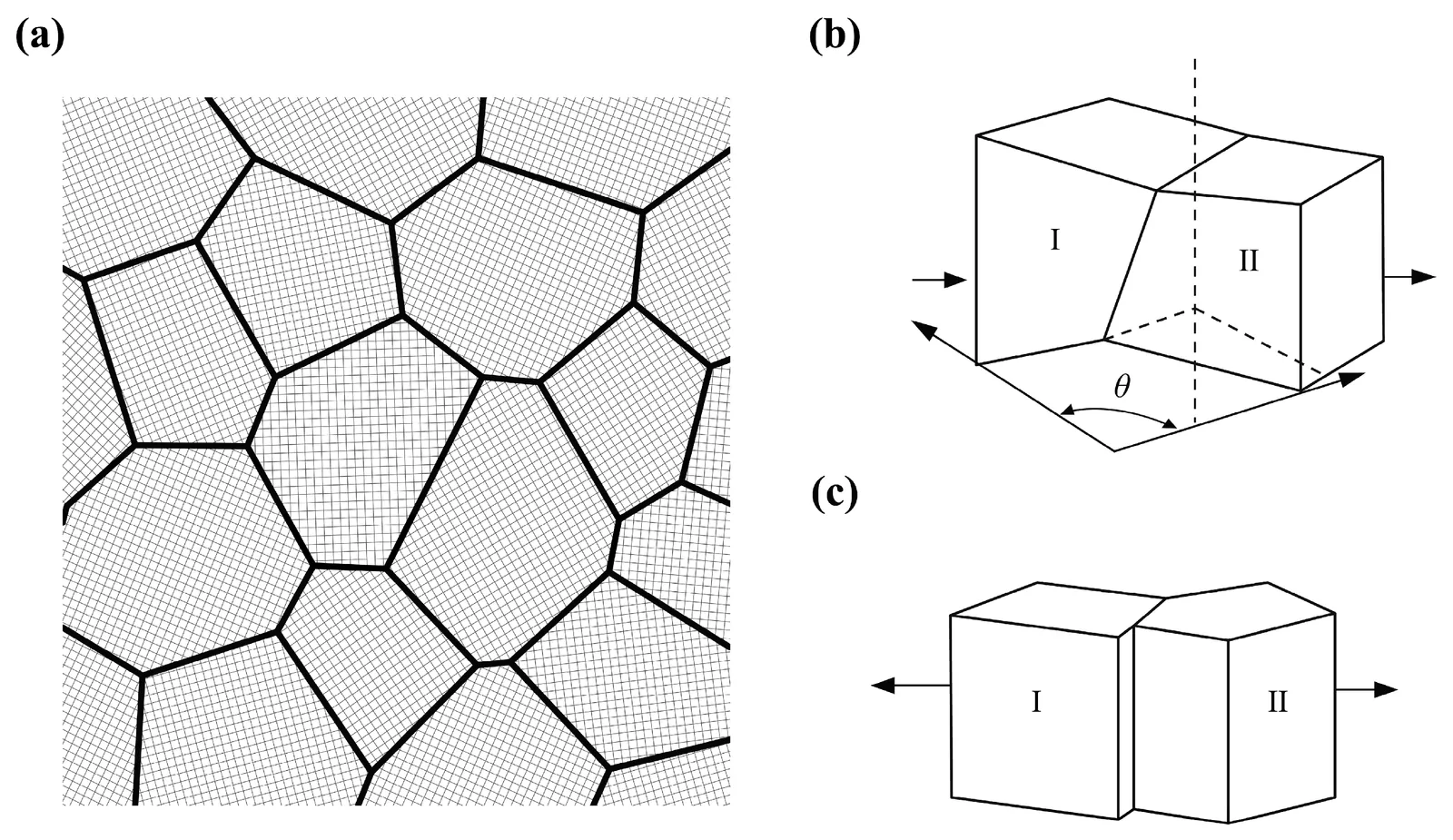
(**a**) Grain boundary structure inside polycrystalline materials; (**b**) tilted grain boundaries; (**c**) twisted grain boundaries. I, crystal I; II, crystal II.

The vast majority of metallic materials used in engineering are polycrystalline, and the interfaces between adjacent grains are referred to as grain boundaries (GBs) [5]. As a common type of two-dimensional defect in polycrystals, grain boundaries possess a disrupted periodicity in atomic arrangement, and this atomic configuration, distinct from that of the grain interior, can significantly influence various physicochemical properties of materials, such as mechanical performance and corrosion resistance [6,7,8]. Grain boundary segregation engineering, by doping with specific elements to regulate the segregation behavior at grain boundaries, can modify both the grain boundary structure and chemical composition, thereby providing an important pathway for developing materials with superior comprehensive properties [9]. The atomic structure of a grain boundary is not fixed but is determined collectively by geometric factors including the orientation relationship between adjacent grains, the rotation axis, the misorientation angle, and the grain boundary plane orientation, as illustrated in Figure 2. A complete description of a grain boundary generally requires five geometric degrees of freedom: three parameters characterizing the orientation difference between the two grains and two parameters defining the grain boundary plane orientation. Based on the relative rotation of adjacent grains, grain boundaries can be classified into tilt grain boundaries, twist grain boundaries, and mixed grain boundaries; according to the symmetry and lattice matching across the interface, they can be further categorized into symmetric or asymmetric grain boundaries and coherent or incoherent grain boundaries.

Grain boundary strength reflects the resistance of an interface to separation and intergranular fracture. Introducing appropriate solute elements into grain boundaries can either enhance or reduce grain boundary cohesion, and proper grain boundary strengthening contributes to improvements in tensile strength, fracture toughness, and fatigue performance [10,11,12]. Before discussing solute effects, it is necessary to clarify two closely related but distinct quantities. Grain boundary cohesion refers to the resistance of the boundary to separation, quantified by the work of separation—the energy required to cleave the boundary into two free surfaces; a higher work of separation corresponds to stronger cohesion. The strengthening energy is defined explicitly as the segregation-induced reduction in the work of separation:

(1) $$\Delta E=Wsep\left(clean\right)-Wsep\left(segregated\right)$$

where W*sep*(*clean*) is the work required to cleave a clean grain boundary into two free surfaces, and W*sep*(*segregated*) is the corresponding work after the solute has segregated to the boundary. Equivalently, in the Rice–Wang framework, the strengthening energy can be expressed in terms of the solute segregation energies:

(2) $$\Delta E=Eseg\left(GB\right)-2Eseg\left(FS\right)$$

where E*seg*(*GB*) and E*seg*(*FS*) are the segregation energies of the solute at the grain boundary and at the free surface, respectively (both conventionally negative, with a more negative value indicating stronger segregation). With this definition, a positive ΔE means that segregation lowers the work of separation—i.e., the boundary becomes easier to cleave—and the solute therefore embrittles (weakens) the boundary; a negative ΔE means the reverse, i.e., the solute strengthens the boundary [13]. It should be noted that the term “strengthening energy” may be misleading, because a positive value actually indicates weakening; throughout this review, “strengthening energy” is used strictly in this Rice–Wang sense.

The Rice–Wang model assesses whether a solute strengthens or embrittles a grain boundary by comparing its segregation preference between the grain boundary and the free fracture surface [14,15]. For uranium specifically, recent DFT calculations on non-metallic segregation at uranium grain boundaries [16] have quantified this strengthening energy for C, N, O, Si, P, and S. All six solutes yield positive strengthening energies, indicating that they intrinsically weaken the grain boundary. Si produces the largest value, 2.9 eV, driven mainly by its 2.1 eV mechanical contribution, whereas C, N, and O contribute much smaller values (0.4–0.5 eV). More importantly, when hydrogen is co-segregated, a single hydrogen atom alone contributes a strengthening energy of 3.37 eV—the largest among all dopants—so that hydrogen itself is the most detrimental species to uranium grain boundary cohesion.

However, the Rice–Wang model is inherently a static energy-based evaluation framework and cannot capture the dynamic changes in atomic structure during deformation, nor can it mechanistically elucidate the fracture strength of grain boundaries and their response behavior under applied stress. In contrast, the second approach employs density functional theory (DFT) or molecular dynamics (MD) simulations to perform computational tensile tests that mimic the mechanical behavior of materials under loading, thereby predicting their macroscopic properties [17,18,19]. Among these, first-principles methods do not rely on empirical potentials and are better suited for analyzing the influence of different dopant elements on grain boundary bonding and mechanical properties.

Grain boundaries are also preferential regions for solute and impurity segregation. Owing to the irregular atomic sites, local strain, and distinctive chemical environment at grain boundaries, certain elements exhibit higher stability at grain boundaries than in the grain interior, leading to localized solute enrichment. Grain boundary segregation engineering, by controlling the type, concentration, and distribution of solutes at grain boundaries, actively modifies the grain boundary structure and chemical composition, thereby offering an important route for optimizing the properties of polycrystalline materials [20,21,22,23]. Whereas conventional grain boundary engineering primarily focuses on adjusting the fraction of special grain boundaries, grain boundary segregation engineering places greater emphasis on regulating interfacial chemistry at the atomic scale. Solute segregation not only alters the grain boundary energy and cohesive strength but may also influence defect formation, atomic diffusion, and interfacial reactivity, thereby exerting significant effects on the mechanical properties and corrosion resistance of materials.

Experimental characterization and theoretical modeling are the two principal approaches for investigating grain boundary segregation and its underlying mechanisms. Transmission electron microscopy and atom probe tomography can characterize grain boundary structures and elemental enrichment features; however, the changes in electronic structure, chemical bonding, and interfacial strength induced by segregation still require atomic-scale simulations for mechanistic interpretation. Consequently, combining experimental characterization with computational methods such as first-principles calculations and molecular dynamics has become an essential strategy for studying grain boundary structure, strength, and segregation behavior.

### 2.3. Experimental Evidence of Intergranular Hydriding

In practical application scenarios, no metallic material can consist of a perfect single-crystal structure; the metallic matrix invariably contains defects to varying degrees. As a typical planar defect, grain boundaries possess relatively high energy and thus exhibit properties distinct from those of the matrix, rendering them susceptible to intergranular corrosion. Hydrogen tends to preferentially accumulate at grain boundaries, leading to the initial nucleation of hydriding corrosion. The following section reviews the experimental research progress on grain-boundary-related hydriding corrosion of uranium.

**Figure 3:**
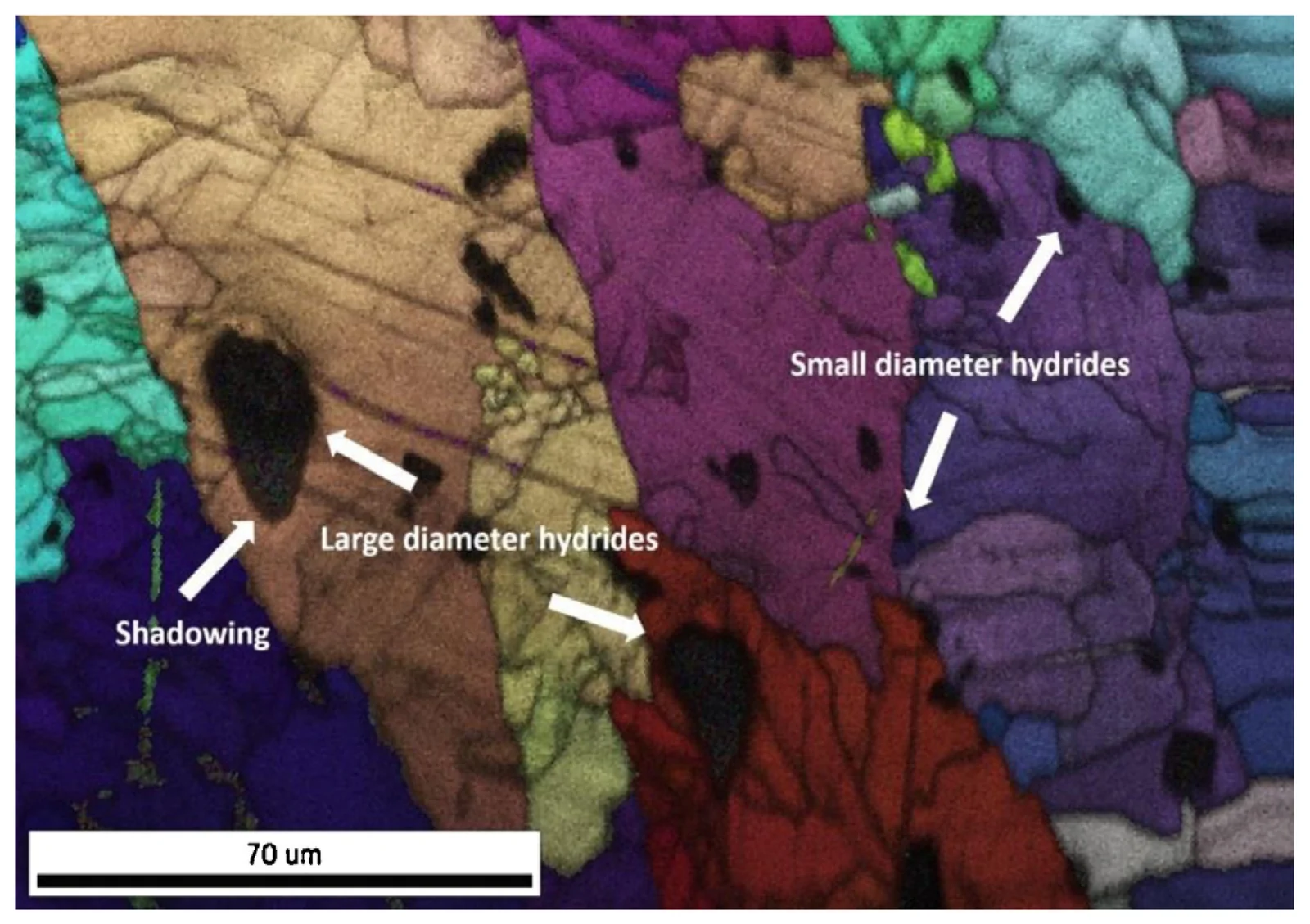
Electron back-scattered diffraction (EBSD) map of an electropolished and spot-hydrided uranium surface. Hydride spots belonging to both the large-diameter and the small-diameter families are located mainly around grain boundaries and twin boundaries. Reproduced from Ref. [25] under the terms of the Creative Commons CC BY 4.0 license.

Numerous studies have revealed that hydrides tend to preferentially nucleate and grow at these planar defects, and the underlying mechanism is closely related to the distinctive energy states, diffusion pathways, and stress concentration effects at the defect sites. Balooch et al. [24] employed temperature-programmed desorption (TPD) and atomic force microscopy (AFM) to investigate the interactions of uranium with hydrogen gas and water vapor at different temperatures. AFM observations revealed that uranium hydride formed preferentially at grain boundaries. They proposed that the preferential formation of hydrides at grain boundaries arises from two complementary effects that are not contradictory: grain boundaries act as fast diffusion channels, along which hydrogen atoms migrate more readily than in the lattice interior, while simultaneously providing deeper binding sites (i.e., a higher binding energy) that trap hydrogen atoms and prolong their residence time. The former facilitates the transport of hydrogen to the boundaries, whereas the latter promotes its accumulation there, and together they lead to local hydrogen enrichment and preferential hydride nucleation at grain boundaries. Banos et al. [25] subsequently provided statistically based evidence for this behaviour by examining uranium surfaces prepared through four different routes—coarse finishing, fine polishing, nitric acid etching, and electropolishing—and then hydriding them simultaneously under identical conditions. As shown in Figure 3, the electron back-scattered diffraction (EBSD) map of the electropolished and spot-hydrided surface reveals that hydride spots belonging to both the large-diameter and the small-diameter families are distributed predominantly around grain boundaries and twin boundaries, whereas the grain interiors remain essentially unattacked. The same spatial preference persisted across all four surface finishes, although the number of nucleation sites per unit area differed considerably, being lowest on the electropolished surface where the oxide layer was thinnest. These observations indicate that the preferential nucleation of uranium hydride is governed by the crystallographic defect network rather than by the surface preparation itself, and that grain boundaries and twin boundaries act simultaneously as fast diffusion pathways and as trapping sites for hydrogen.

Bingert et al. [26] employed automated electron back-scattered diffraction (EBSD) to characterize the local crystallographic orientation of hydrided uranium samples, including orientation mapping, deformation twin identification, and grain boundary analysis. The experimental results revealed that hydrides formed preferentially at high-angle grain boundaries, low-angle grain boundaries, and twin boundaries of two systems.

**Figure 4:**
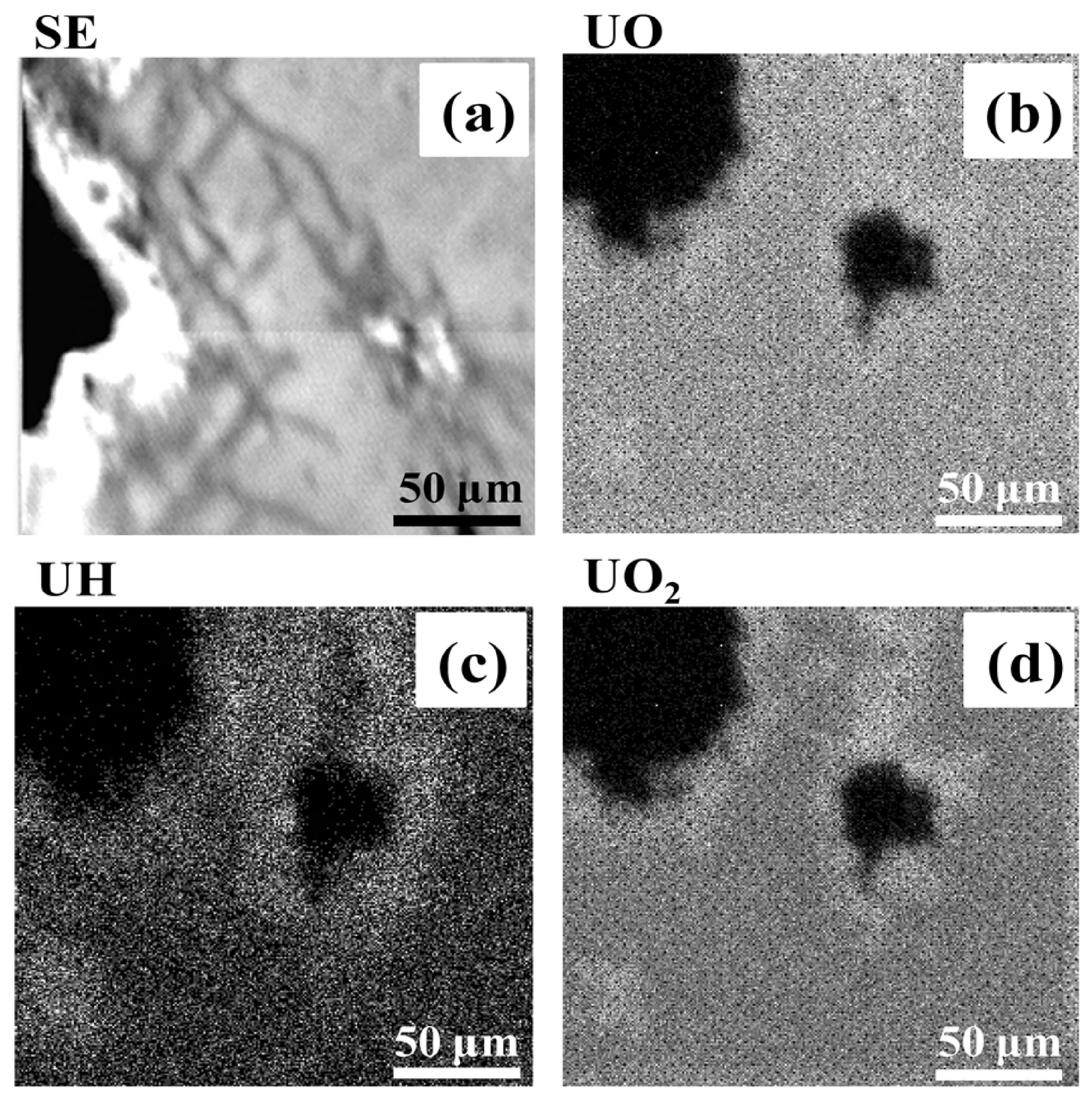
Secondary ion imaging of hydride growth sites on a polished uranium surface. (**a**) Secondary electron image of a 200 × 200 μm region showing hydriding craters; (**b**–**d**) ToF-SIMS images of the same region for the UO^+^, UH^+^, and UO_2_^+^ secondary ions, respectively. The UH^+^ map shows that hydrogen is enriched in an extended zone surrounding each crater. Reproduced from Ref. [28] under the terms of the Creative Commons CC BY 4.0 license.

Scott et al. [27] investigated the relationship between UD_3_ precipitate sites on uranium surfaces and metal grain boundaries using secondary ion mass spectrometry (SIMS) and focused ion beam (FIB) techniques. Their experiments revealed that a large number of growth sites were distributed exclusively along metal grain boundaries in a linear pattern. Through SIMS positive ion mapping, they confirmed that the growth chains observed along the metal grain boundaries were UD_3_ precipitates. Ji et al. [28] subsequently mapped the local hydrogen distribution around individual hydride craters by ToF-SIMS; as shown in Figure 4, the UH^+^ image reveals an extended hydrogen-enriched zone surrounding each crater, which they identify as a hydrogen-diffused transition region. Jones et al. [29] employed focused ion beam milling and high-resolution secondary electron imaging to investigate hydride growth sites on the sample surface, and used EBSD to analyze the metal grain orientation in the corroded regions. EBSD analysis revealed that, in the FIB-etched area of a thoroughly polished and hydriding-corroded uranium surface, hydride growth nucleated predominantly (greater than 90%) at grain boundaries. In particular, hydrides formed at 320 °C tended to nucleate at grain boundaries with high lattice misorientation angles.

**Figure 5:**
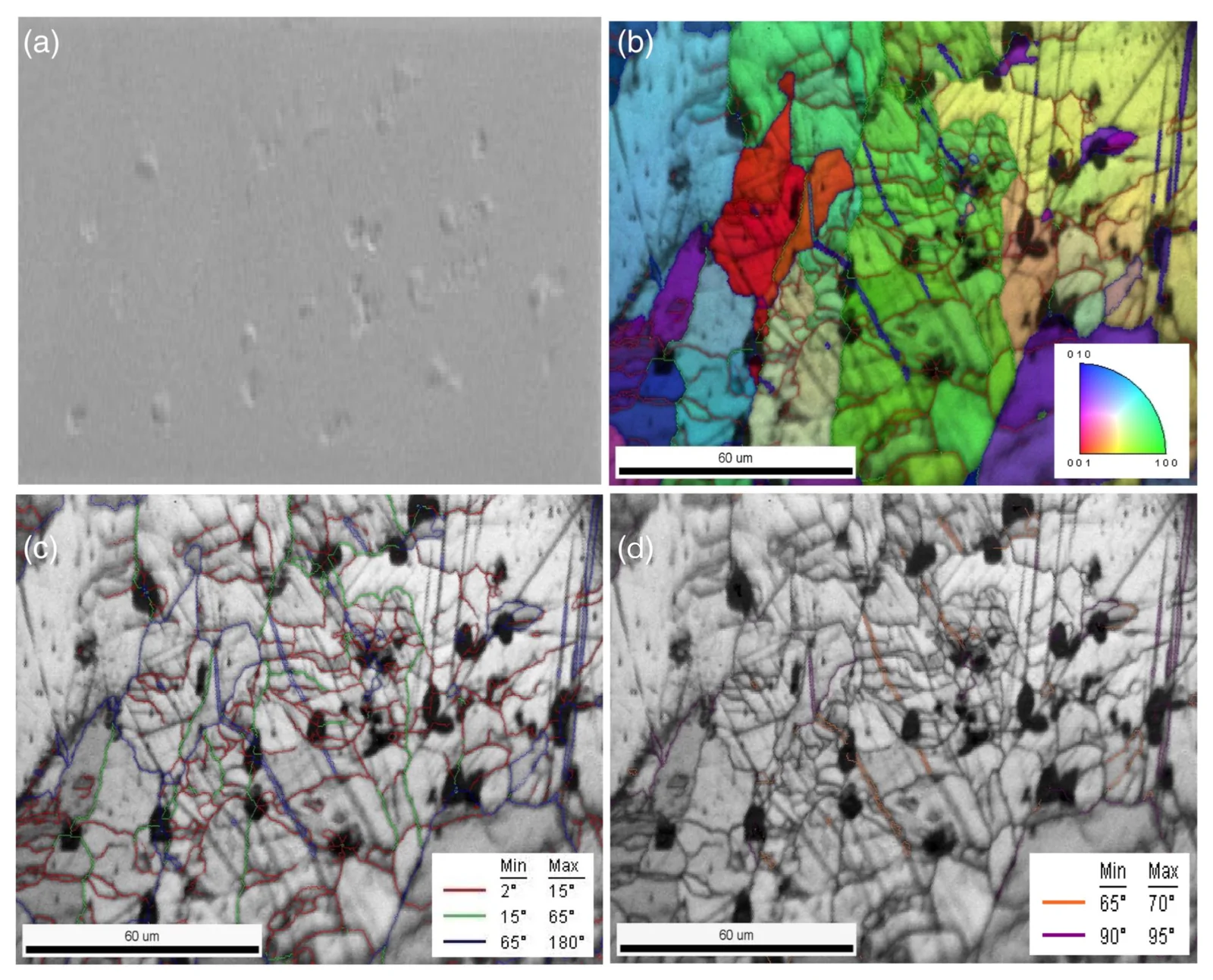
Electron-scale EBSD map of uranium hydride region: (**a**) SE image; (**b**) crystallographic orientation map; (**c**) misorientation boundary map; (**d**) twin boundary map [30].

Banos et al. [30] performed a statistical analysis of uranium hydride nucleation sites using EBSD; an electron-scale EBSD map of the hydrided uranium region is presented in Figure 5. Through scanning electron microscopy (SEM) and secondary ion mass spectrometry analysis, they found that hydrides formed predominantly around grain boundaries and twin boundaries. EBSD analysis showed that over 90% of hydride sites were associated with grain boundaries and twin boundaries, especially in the vicinity of grains with high rotational mismatch. These data support the view that hydride nucleation sites are strongly influenced by the microstructure of the metal surface.

Integrating experimental results across different scales from AFM, EBSD, SIMS, and FIB, it can be concluded that preferential grain boundary hydriding is not an isolated morphological phenomenon but rather a universal microscopic characteristic of the hydriding corrosion of metallic uranium. Nevertheless, existing experiments have primarily established the spatial correlation between hydrides and grain boundaries. A unified atomic-scale understanding remains lacking with respect to why different grain boundary structures exhibit distinct hydrogen trapping capabilities, whether grain boundaries act as hydrogen traps or fast diffusion channels, and how hydrogen enrichment at grain boundaries further evolves into UH_3_ nucleation. Therefore, elucidating the intrinsic connections among grain boundary structure, hydrogen segregation, and hydride nucleation is central to understanding the localized hydriding corrosion mechanism of metallic uranium.

## 3. Role of Grain Boundaries in the Hydriding Corrosion of Metallic Uranium

### 3.1. Hydriding Corrosion Process on the Surface of Metallic Uranium

Owing to its exceptionally high oxygen affinity, metallic uranium spontaneously forms an oxide film on its surface under ambient conditions [31]. This in situ-grown oxide layer serves as a critical interface that participates in and regulates the entire process of hydriding corrosion initiation and development. It exerts significant influence on the onset time, spatial distribution, and reaction kinetics of corrosion, and constitutes a central element in understanding the heterogeneous characteristics of the uranium–hydrogen reaction [32].

**Figure 6:**
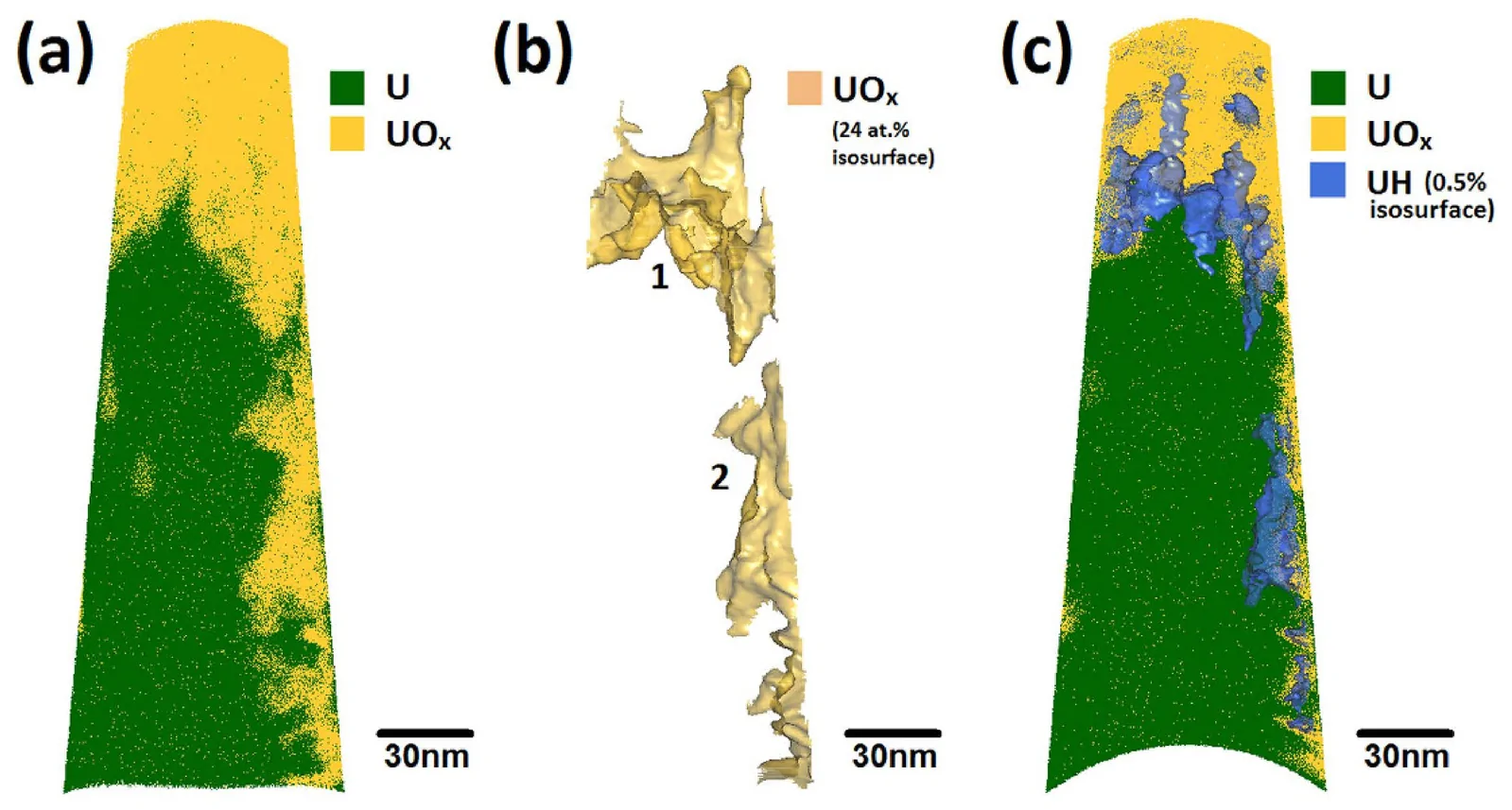
(**a**) An atom probe map of a tip extracted from a uranium sample exposed to air for approximately 1 h, showing U and UOx ions in green and yellow/orange, respectively. The original surface of the uranium is located at the top of the specimen. (**b**) A 24 at.% UO/UO2 isosurface indicating two oxide regions on the specimen; (marked 1) at the original surface and (marked 2) generated on the side of the specimen during sample preparation. (**c**) The same atom map as in (**a**), but with an isoconcentration surface indicating 0.5 at.% UH in blue to reveal the locations where hydride ions are detected. For (**c**), the front face of the dataset is cropped away to show a cross-section of the middle of the specimen. Adapted from Martin et al. [33] under the terms of the Creative Commons Attribution 4.0 licence.

Its thickness, composition and continuity are, however, far from uniform. Atom probe tomography of a polished uranium surface exposed to moist air for only about one hour has revealed a markedly corrugated oxide/metal interface, with an oxygen content of approximately 60 at.% within the oxide and with discrete, unevenly distributed oxide regions (Figure 6) [33]. Such local variations in oxide thickness and stoichiometry are expected to translate directly into a spatially non-uniform barrier to hydrogen uptake, so that hydrogen preferentially penetrates the thinner or more defective parts of the film. This provides a natural microscopic origin for the pronounced site selectivity of hydride nucleation, and indicates that the incubation period and the spatial distribution of corrosion pits cannot be described by a single average oxide thickness.

The oxide layer acts as a diffusion barrier that suppresses the reaction kinetics, and the presence of the surface oxide layer significantly delays the onset of hydriding corrosion. Hydrogen gas (H_2_) must first dissociate into hydrogen atoms (H) on the surface, after which the H atoms must traverse this solid layer to reach the metallic substrate. Consequently, within a certain thickness range, the oxide layer plays the role of a kinetic barrier. Studies have shown that a thicker oxide layer leads to a longer diffusion path and greater resistance for hydrogen atoms, resulting in a correspondingly prolonged incubation period and a markedly reduced density of corrosion nucleation sites per unit area [34].

Early kinetic measurements also confirmed that the incubation period increases with oxide layer thickness and the nucleation rate decreases accordingly [35]. However, this barrier effect has an upper limit. When the oxide layer grows excessively thick, for example beyond approximately 300 nm, the accumulated internal stress arising from the mismatch in thermal expansion coefficients between the oxide and the substrate—which becomes significant upon temperature changes, for example during thermal cycling between room temperature and elevated service temperatures—can induce cracking or spallation of the oxide film. These defects provide fast diffusion pathways for hydrogen, causing the effect of further increasing the thickness to prolong the incubation period to rapidly diminish or even vanish.

Oxide layer defects can serve as preferential nucleation sites for corrosion. Hydriding corrosion manifests itself in the form of pitting, with nucleation sites exhibiting high spatial selectivity, which is directly related to the microscopic inhomogeneity of the oxide layer. Around mechanical scratches and hard inclusions such as carbides, stress concentration often results in incomplete oxide coverage, the presence of microcracks, or the formation of locally weakened regions, leading to the predominant distribution of hydrides in scratched surface areas. These regions provide low-resistance pathways for hydrogen permeation, thereby becoming preferential sites for corrosion nucleation [25,36]. For instance, the mismatch between carbide inclusions, the metallic matrix, and the oxide layer can provide a shortcut for hydrogen diffusion, promoting preferential hydride nucleation and growth at these locations [37].

Although experimental methods remain a traditional and effective means of studying the corrosion processes of uranium and uranium alloys, they possess inherent limitations in revealing microscopic reaction mechanisms at the atomic and even electronic scales. The origin of surface corrosion typically begins with the adsorption, dissociation, and subsequent atomic diffusion of small gas-phase molecules (such as H_2_, O_2_, and H_2_O) on the material surface. Owing to the transient and microscopic nature of these initial processes, conventional experimental methods are unable to perform in situ observation and precise analysis. To overcome this bottleneck, theoretical computational approaches based on quantum mechanics, particularly first-principles calculations, have become indispensable for in-depth investigations of the intrinsic properties and microscopic mechanisms of materials [38], and a series of advances have already been made in the study of uranium surface corrosion mechanisms.

The hydriding corrosion of metallic uranium begins with the behavior of hydrogen atoms on the surface oxide. Goel et al. [39] employed density functional theory to investigate hydrogen adsorption on surfaces containing grain boundaries, and found that grain boundary sites on the uranium dioxide surface exhibit lower adsorption energies, indicating that hydrogen adsorbs more readily at surface grain boundary sites. However, the subsequent interactions of hydrogen within uranium dioxide grain boundaries have not been reported in the open literature. Studies on uranium dioxide grain boundaries have primarily focused on energetic properties and fission product segregation. Yang et al. [40] employed molecular statics/dynamics simulations in conjunction with three different empirical potentials to investigate the stability of tilt grain boundaries in uranium dioxide (UO_2_) and their influence on xenon (Xe) segregation behavior. Their results indicate that grain boundaries play a critical role in nuclear materials by affecting the accumulation and release of fission gases such as xenon in uranium oxide.

For uranium surfaces, researchers have systematically investigated the adsorption behavior and interaction mechanisms of various small molecules. For example, simulations of the U–O system by Shuai et al. [41] demonstrated that the 5f electrons of uranium play a dominant role in the formation of U–O bonds. Huang [42] studied the adsorption of H_2_, O_2_, H_2_O, and CO on the α-U(001) surface and found that these molecules tend to adsorb at long-bridge sites, while also revealing the charge transfer and orbital hybridization phenomena occurring during CO adsorption. Liu et al. [43] focused on the migration behavior of oxygen atoms on the α-U(100) surface, and their calculations indicated that while the surface diffusion barrier is relatively low, diffusion into the subsurface is considerably more difficult, thus providing a theoretical explanation for the initial kinetic limitations of surface oxide layer formation. Qin et al. [44] employed density functional theory and ab initio molecular dynamics simulations to study the reaction of γ-U(001) and (110) surfaces upon contact with O_2_. They conducted the first investigation of the adsorption of O atoms and O_2_ molecules on and beneath the (001) and (110) surfaces, providing in-depth insights into the adsorption, dissociation, and diffusion of O_2_ on these surfaces.

**Figure 7:**
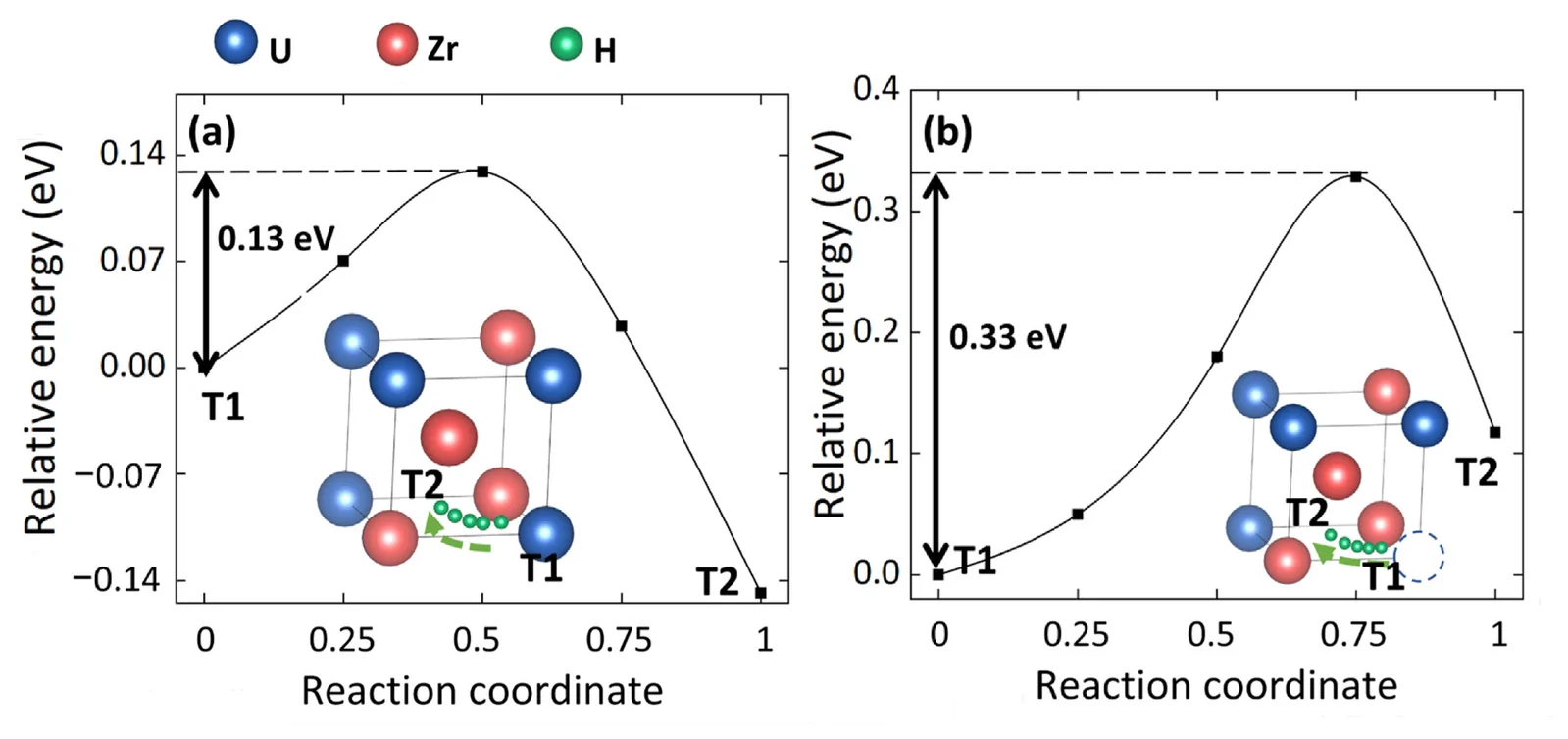
Representative energy barriers for the migration of interstitial hydrogen between adjacent tetrahedral (T) sites in γ(U,Zr), obtained from first-principles calculations: (**a**) perfect lattice; (**b**) lattice containing a uranium vacancy (V_U). The green arrows indicate the hydrogen diffusion path, and T1 and T2 denote the initial and final tetrahedral sites, respectively. The presence of the uranium vacancy markedly raises the migration barrier along the same pathway, indicating that vacancies act as effective hydrogen trapping centres. Reproduced from Huang et al. [47] under the terms of the Creative Commons CC BY 4.0 licence.

In the critical stages of hydriding corrosion, the work of Nie [45] and Yang et al. [46] systematically elucidated the adsorption and dissociation of H_2_ on the α-U(100) surface, as well as the stable adsorption sites and diffusion barriers of hydrogen atoms, thereby providing a detailed energetic picture for understanding the initial steps of hydriding. Yang et al. [46] employed first-principles calculations to conduct the first investigation of the stability, solubility, and diffusion properties of interstitial hydrogen atoms in metallic uranium. Using the climbing image nudged elastic band (CI-NEB) method, they characterized the minimum migration pathways of interstitial hydrogen in the uranium lattice. Beyond the defect-free lattice, however, the influence of lattice defects on hydrogen transport must also be taken into account, since vacancies are inevitably introduced into uranium alloys during irradiation and thermal processing. Figure 7 compares the hydrogen migration barriers between adjacent tetrahedral interstitial sites in γ(U,Zr) calculated for a perfect lattice and for a lattice containing a uranium vacancy [47]. Although the barrier in the perfect lattice is relatively low, the presence of a single uranium vacancy substantially raises the barrier along the same pathway, demonstrating that vacancies act as effective hydrogen trapping centres that retard long-range hydrogen transport. Such defect-controlled trapping provides a plausible atomic-scale explanation for the pronounced spatial heterogeneity of hydride nucleation in uranium alloys and for the strong dependence of hydriding behaviour on thermal and mechanical history. In addition, Li Gan et al. [48,49,50] also performed complementary first-principles studies on the adsorption of molecules such as O_2_, CO, and CO_2_ on the α-U(100) surface, systematically investigating their adsorption configurations and energetic characteristics on the uranium surface, and revealing the interaction mechanisms between the uranium surface and these molecules.

In summary, the transport of hydrogen across the surface oxide layer of metallic uranium is a heterogeneous process governed collectively by the film thickness, defect structure, surface condition, and local microstructure. An intact oxide layer primarily acts as a diffusion barrier, whereas scratches, cracks, weakened regions around inclusions, and potential oxide grain boundary pathways may lead to localized concentration of hydrogen flux and induce preferential hydride nucleation. It is thus evident that an understanding of the initiation process of uranium hydriding corrosion cannot be based solely on the average oxide layer thickness; attention must also be paid to the inhomogeneity of internal defects and local structures within the oxide layer. After penetrating the oxide layer, hydrogen is further subject to trapping and transport by grain boundaries in the metallic matrix, and its segregation and diffusion behavior at grain boundaries in metallic uranium will be discussed in the following section.

### 3.2. Hydrogen Segregation and Diffusion at Grain Boundaries in Metallic Uranium

The aforementioned AFM, EBSD, SIMS, and FIB studies have demonstrated, from both morphological and crystallographic perspectives, that hydrides in metallic uranium exhibit a pronounced spatial correlation with grain boundaries. In some studies, over 90% of hydride sites are located at or near grain boundaries and twin boundaries, and grain boundaries with high misorientation angles generally exhibit greater susceptibility to hydriding [24,25,26,27,28,29,30]. These experimental findings answer the question of “where hydrides preferentially form,” but the questions of why hydrogen enriches at grain boundaries after entering the metallic matrix, why different grain boundaries exhibit distinct hydrogen trapping capabilities, and whether grain boundaries function as hydrogen traps or fast migration channels still require atomic-scale explanation.

**Figure 8:**
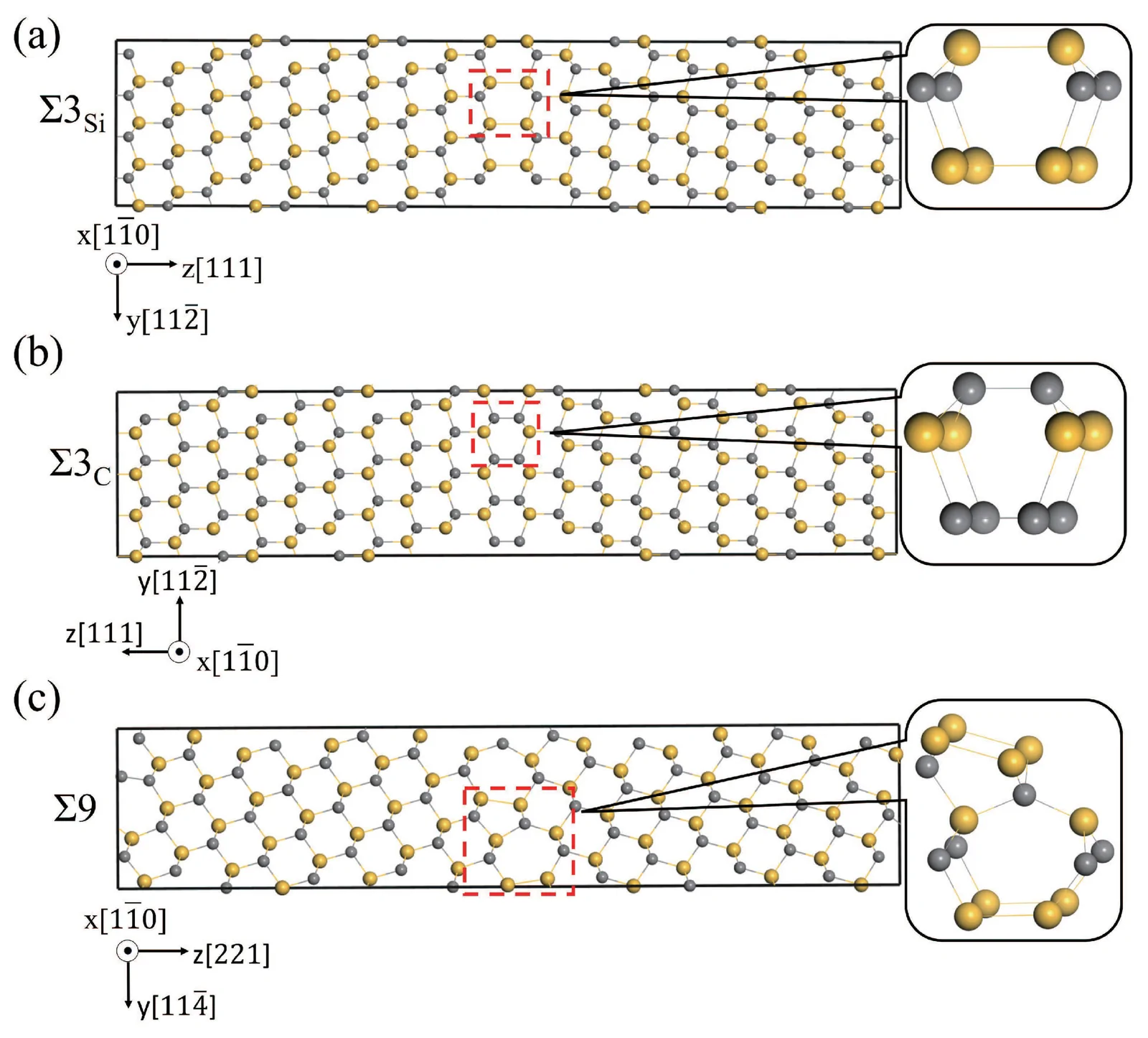
Atomic structures of three representative grain boundaries projected along the [110] direction: (**a**) Σ3Si, (**b**) Σ3C, and (**c**) Σ9. The three boundaries possess distinctly different structural units and boundary-core geometries, so that the excess free volume available at the boundary core—and hence the number and depth of the interstitial sites that can accommodate hydrogen—differs substantially from one boundary to another. Reproduced from Cui et al. [54] under the terms of the Creative Commons CC BY 4.0 licence.

Current theoretical research on uranium grain boundaries has primarily focused on the interaction of grain boundaries with other defects and on properties such as grain boundary migration. Luan et al. [51] employed molecular dynamics simulations to investigate the shock-induced spallation mechanism of polycrystalline uranium, with particular emphasis on the role of grain boundaries. Their simulation results revealed that grain boundaries exert a significant influence on void nucleation and growth; in particular, voids tend to nucleate preferentially at grain boundaries in polycrystalline uranium. The spallation process in polycrystalline uranium proceeds mainly through void growth and coalescence mechanisms, with grain boundaries playing an important role in void nucleation and growth. The spallation strength of polycrystalline uranium is lower than that of single-crystal uranium, and voids form more readily at grain boundary junctions. French et al. [52] studied the mobility and anisotropy of grain boundaries in body-centered cubic (BCC) γ-uranium through molecular dynamics simulations, and found that in γ-uranium, the [110] grain boundaries generally exhibit the highest mobility, which may be related to the atomic planar density. Mahbuba et al. [53] evaluated the anisotropic grain boundary and surface energies of α-uranium via molecular dynamics, providing a comprehensive analysis of the grain boundary and surface energies of α-U and revealing the dependence of interfacial properties on crystallographic orientation and temperature. These calculations show that the grain boundary energy and the interfacial excess volume of α-U are strongly anisotropic, so that boundaries of different character cannot be regarded as equivalent trapping sites for hydrogen. Because openly available first-principles data for hydrogen at uranium grain boundaries remain scarce, the general structural origin of this heterogeneity is illustrated here using a well-characterised model system: Figure 8 compares three prototypical Σ3 and Σ9 boundaries whose structural units and boundary-core free volumes differ markedly from one another [54]. Boundaries with more open cores provide deeper interstitial sites and therefore stronger trapping, whereas densely packed boundaries act as weak traps or even as rapid hydrogen transport channels.

With respect to grain boundaries in uranium metal, related studies have reported the behavior of oxygen atoms therein. Liu et al. [55] employed density functional theory calculations to systematically investigate the interactions of O atoms with different crystallographic planes of α-U and γ-U. as well as the effects of surface strain and twin structures on the initial oxidation of uranium surfaces. The computational results indicate that twin structures have only a minor effect on the initial oxidation of uranium surfaces: they neither substantially suppress nor substantially promote oxidation. In other words, twin boundaries do not markedly alter the intrinsic oxidation behavior of uranium.

**Figure 9:**
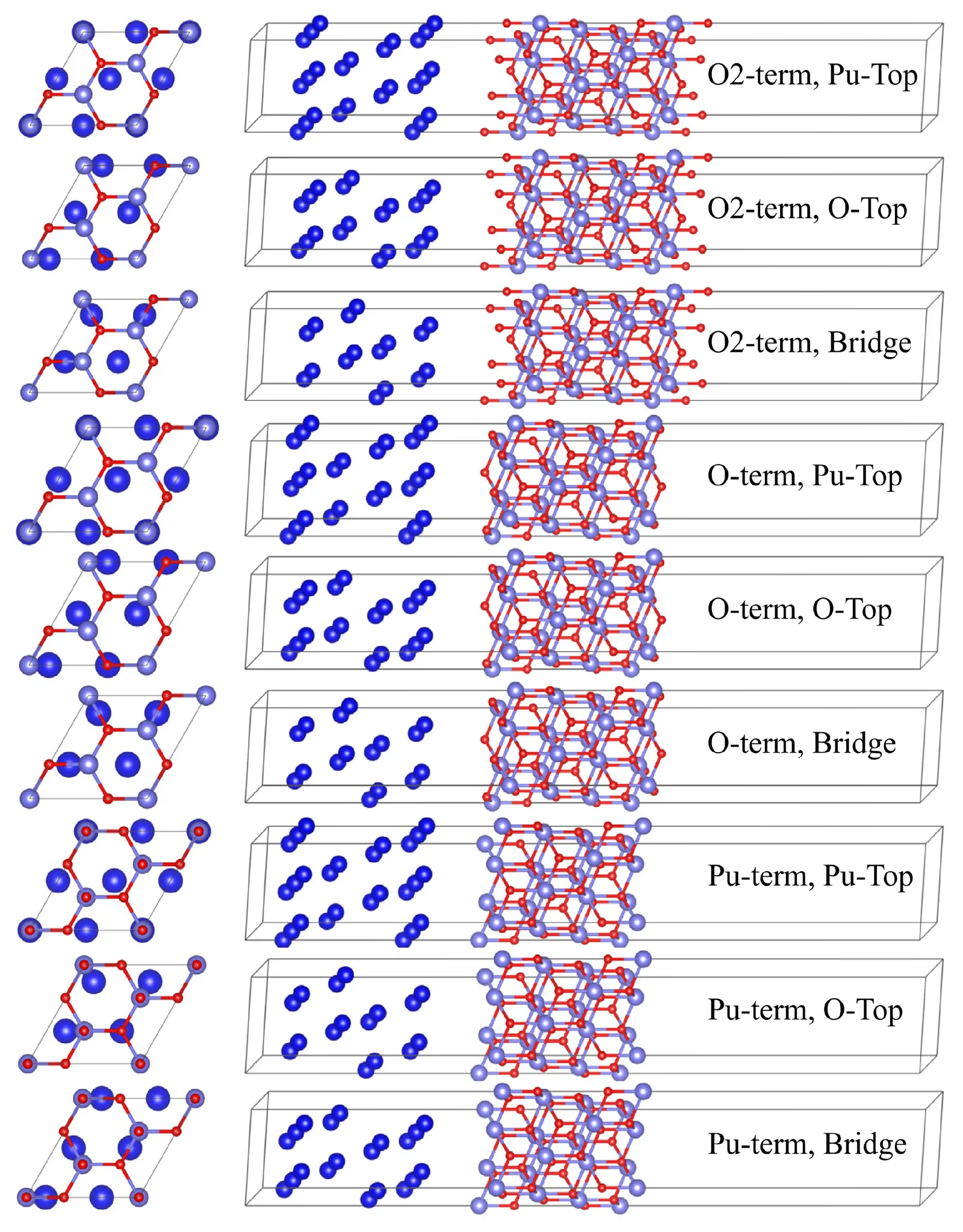
Cross-sectional and front views of a representative actinide metal/oxide interface. For the Pu/PuO_2_(111) interface, nine distinct configurations arise from the combination of three oxide terminations (O^2−^, O^−^, and Pu-termination) and three stacking sequences (‘O-Top’, ‘Pu-Top’, and ‘Bridge’), with a lattice misfit of only about 1%. The blue, cyan, and red spheres denote metallic cations, oxidised cations, and oxygen atoms, respectively. The termination determines the interfacial separation and the work of adhesion, whereas the stacking sequence has only a minor effect. Such structural multiplicity is a general feature of metal/oxide interfaces and governs the local atomic environment and the interstitial space available at the interface. Reproduced from Qiu [59] under the terms of the Creative Commons CC BY 4.0 licence.

There are relatively few openly published studies on hydrogen atoms at uranium grain boundaries. At material interfaces, related research has reported the behavior of hydrogen at two-phase interfaces and oxide interfaces. Wang et al. [56] combined experiments and simulations to reveal the characteristics of hydrogen distribution in dual-phase uranium, demonstrating that the body-centered cubic lattice of γ-U and alloyed Nb atoms suppresses hydrogen distribution on the γ-U side. This work highlights the important influence of lattice structure on hydrogen accumulation in metals and provides new insights for improving the hydrogen corrosion resistance of uranium alloys. Wang et al. [57] systematically investigated the distribution of hydrogen at the α-U/UO_2_ interface by combining PBE and PBE+U methods. They found that hydrogen atoms initially tend to accumulate in the interfacial plane region, forming two hydrogen atomic layers. After saturation, subsequent hydrogen atoms preferentially reside on the α-U side, revealing the complex hydrogen distribution characteristics at the α-U/UO_2_ interface and providing an important reference for further studies on the hydrogen-induced corrosion of uranium. Jin et al. [58] employed first-principles theory to study the hydrogen dissolution characteristics at the (130)/[001] grain boundary in α-U metal. Their results show that the dissolution energy of hydrogen atoms is lowest at the (130)/[001] grain boundary and increases with distance from the grain boundary. A metal/oxide interface, however, is not a simple geometric plane but a structurally complex region in its own right. Taking the strongly correlated Pu/PuO_2_(111) interface as a representative case, nine distinct interfacial configurations can be constructed by combining three oxide terminations with three stacking sequences, all with a lattice misfit of about 1% (Figure 9) [59]. The termination—that is, whether the oxide side is O^2−^-, O^−^-, or Pu-terminated—primarily controls the interfacial separation and the work of adhesion, whereas the stacking sequence plays only a minor role. Such structural multiplicity means that the interface provides its own set of interstitial sites and a locally modified bonding environment, rather than being a smooth continuation of either bulk phase. This structural picture offers a natural explanation for the hydrogen accumulation behaviour reported for the α-U/UO_2_ interface, where hydrogen preferentially occupies the interfacial plane and forms two hydrogen-rich atomic layers [59].

Overall, existing experiments have clearly established that grain boundaries are preferential sites for hydride formation in metallic uranium, and micro-scale calculations further indicate that this phenomenon originates from hydrogen segregation driven collectively by local grain boundary structure, elastic distortion, and electronic interactions. Different types of grain boundaries may possess distinct hydrogen trapping strengths and migration capacities, and grain boundaries may also exhibit different characteristics—ranging from thermodynamic traps to fast transport channels—depending on the hydrogen concentration and local structural variations. A key task for future work is to systematically compare hydrogen segregation energies, diffusion barriers, and electronic structure changes for representative α-U general grain boundaries, low-Σ grain boundaries, and twin boundaries, while further considering the coupling effects of vacancies, solutes, and stress, thereby establishing a quantitative relationship linking grain boundary structure, hydrogen segregation, grain boundary diffusion, and local supersaturation.

### 3.3. Summary

Existing studies have confirmed, at both the experimental and atomic scales, that the surface oxide layer and grain boundaries jointly govern the localized initiation of hydriding corrosion in metallic uranium. Experimental characterizations have relatively clearly elucidated the effects of oxide layer thickness, cracks, and inclusions on the incubation period and nucleation sites, and have also confirmed that hydrides form preferentially near metal grain boundaries and twin boundaries [24,25,26,27,28,29,30]. Building upon this foundation, first-principles studies have begun to elucidate the stabilization mechanisms of hydrogen at UO_2_ grain boundaries and near α-U grain boundaries from the perspectives of local geometric distortion, coordination environment, and electronic interactions. Recent studies have shown that various UO_2_ grain boundaries possess stronger hydrogen binding capabilities than the perfect lattice [39], indicating that oxide grain boundaries may serve as preferential hydrogen trapping regions. Calculations on α-U twin boundaries have also shown that hydrogen trapping is related not only to local free volume but also to the orbital interactions between hydrogen and uranium atoms [58].

However, the current understanding remains largely at the level of “hydrogen tends to be stable at grain boundaries.” Low adsorption energies or segregation energies merely indicate that grain boundaries possess a thermodynamic tendency to trap hydrogen, but do not directly prove that they serve as fast diffusion channels. Existing studies still lack a systematic comparison of the continuous migration pathways and associated barriers for hydrogen transport from the oxide surface through UO_2_ grain boundaries and the UO_2_/α-U interface, and ultimately into the metallic grain boundaries. Nor have they adequately addressed whether different grain boundaries behave as deep traps or as transport channels along the interface. Moreover, most calculations employ ideal, clean, and low-hydrogen-concentration models, with insufficient consideration of vacancies, solute segregation, stress, and multi-hydrogen interactions in real grain boundaries. Consequently, the transition from single-hydrogen segregation to local supersaturation and ultimately to UH_3_ nucleation still lacks a direct description.

In recent years, multi-phase-field models have incorporated hydrogen diffusion, grain boundary effects, hydride growth, and the mechanical response of the oxide film into a unified framework, enabling the reproduction of complex morphologies such as multi-site corrosion and intergranular hydrides [32]. Further corrosion–fracture coupled simulations have shown that the stress induced by hydride growth can drive the oxide film to crack along grain boundaries, and these cracks in turn create new hydrogen transport pathways, thereby generating a positive feedback loop that accelerates corrosion. However, the grain boundary diffusivity, segregation coefficients, and fracture parameters in such mesoscopic models still rely heavily on assumptions or empirical fitting, and a reliable connection with atomic-scale calculations and experimental measurements has yet to be established.

Therefore, future research should shift from merely demonstrating that “grain boundaries can trap hydrogen” toward establishing a quantitatively verifiable cross-scale mechanism: systematically comparing hydrogen segregation energies and migration barriers in general grain boundaries, low-Σ grain boundaries, and twin boundaries; considering the synergistic effects of vacancies, alloying elements, non-metallic impurities, and stress; and transferring atomic-scale parameters into reactive molecular dynamics and phase-field models. Experimentally, it is necessary to employ isotope tracing, quasi-in situ characterization, and three-dimensional interface analysis to verify the actual migration sequence of hydrogen among oxide grain boundaries, metal–oxide interfaces, and metallic grain boundaries. Only through such efforts can it be further clarified which grain boundaries are most susceptible to hydrogen enrichment, and how localized hydriding corrosion can be suppressed through the design of interfacial structure and chemical composition.

The atomic-scale picture established above has direct implications for engineering practice. Because localized hydriding initiation is governed by the coupled behavior of the oxide layer and grain boundaries, any strategy that alters the local chemistry or structure of these interfaces can, in principle, modulate the corrosion susceptibility of the material. Alloying elements and impurities represent the most practical and controllable levers available at the engineering scale, as their segregation to grain boundaries directly reshapes the same interfacial electronic structure, cohesive strength, and hydrogen-trapping behavior identified at the atomic level. The following section therefore examines how metallic alloying elements and non-metallic impurities regulate grain boundary hydriding corrosion, connecting these atomic-scale mechanisms to compositional and processing design.

## 4. Regulation of Grain Boundary Hydriding Corrosion by Alloying Elements and Impurities

### 4.1. Role of Metallic Alloying Elements

Alloying is a key approach for enhancing the comprehensive properties of metallic materials. Elements such as chromium (Cr), molybdenum (Mo), niobium (Nb), titanium (Ti), and zirconium (Zr) can form continuous solid solutions with uranium in the high-temperature γ-phase region [60]. The introduction of such alloying elements fundamentally alters the crystal structure, phase stability, and defect chemistry of the matrix, thereby exerting a profound influence on the thermodynamics and kinetics of hydriding corrosion. Investigating the mechanisms by which alloying elements operate is essential for enhancing the hydriding corrosion resistance and mechanical properties of uranium materials through compositional design.

The content of alloying elements is the most direct parameter for regulating hydriding behavior, and current research has focused on several typical alloy solutes, such as uranium–niobium alloys. Studies have shown that in quenched U–Nb alloys with Nb contents below 7 wt.%, the incubation period of the hydriding reaction is significantly prolonged and the overall reaction rate decreases markedly with increasing Nb content [61]. When the Nb content reaches approximately 5 wt.%, the hydriding corrosion rate is already substantially reduced compared with low-Nb alloys; if the Nb content continues to increase, the alloy even tends to resist the occurrence of the hydriding reaction [61]. This phenomenon is closely related to the ability of Nb to stabilize the high-temperature γ phase (body-centered cubic structure) and suppress its transformation to the α phase (orthorhombic structure). The γ phase possesses higher crystal symmetry and a more open crystal structure, which may be unfavorable for rapid hydrogen diffusion or hydride nucleation. Meanwhile, dissolved Nb atoms may modify the energy states at defects such as grain boundaries through chemical bonding, thereby influencing the segregation behavior of hydrogen.

With increasing Nb content in U–Nb alloys, the hydriding corrosion morphology gradually transitions from random pitting similar to that of pure uranium to directional pit propagation with pronounced orientation. In low-Nb-content alloys (e.g., <3 wt.%), the corrosion pit morphology is similar to that of pure uranium, but with a larger critical rupture size; whereas in high-Nb-content alloys, the propagation direction of the corrosion sites may be strongly constrained by the specific crystallographic orientation or microstructural features of the matrix [61]. This morphological evolution directly reflects the modulation of hydride nucleation sites and growth anisotropy by alloying, and the primary reason lies in the fact that alloying elements modify the grain boundary or interface properties, thereby influencing the stress field and crack propagation path associated with hydrides.

**Figure 10:**
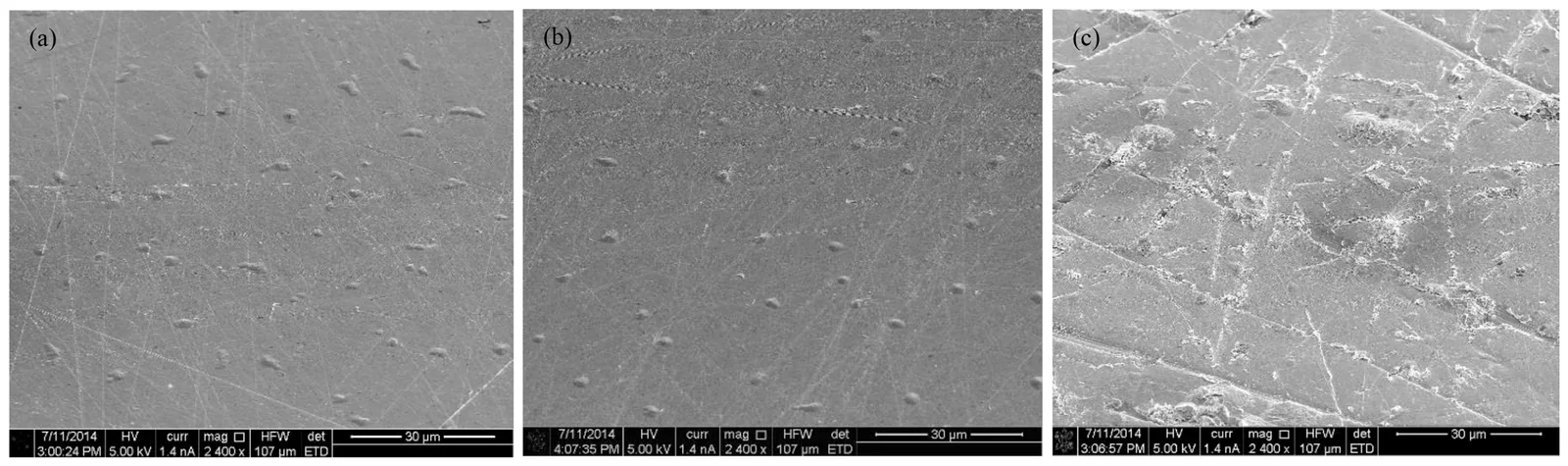
Dependence of early-stage hydriding behaviour on the microstructure established by thermal and mechanical treatment. Secondary electron (SE) images of Magnox-U surfaces after partial reaction under 500 mbar (50 kPa) D_2_ at 190 °C: (**a**) as-cast; (**b**) thermally annealed (550 °C, 140 h); (**c**) work-hardened. Although the three samples have the same composition, they exhibit markedly different hydride site densities and hydride morphologies. Adapted from Banos et al. [62] under the terms of the Creative Commons Attribution 4.0 licence.

The hydriding corrosion behavior of uranium alloys is not solely determined by composition but also depends strongly on the heat treatment processes that govern their microstructure. For instance, in U–13at.%Nb alloys, different aging treatments lead to variations in the morphology, distribution, and chemical composition of the decomposition products of the metastable γ phase. These distinct microstructures exhibit markedly different hydrogen solubility, diffusion coefficients, and defect densities, resulting in significant differences in the incubation period, nucleation density, and growth kinetics of surface hydriding corrosion. This indicates that the ultimate effect of alloying elements is realized through the specific microstructure formed by their synergistic interaction with the heat treatment process, including grain boundary structures and precipitate characteristics. The controlling role of microstructure is not restricted to alloyed material. As shown in Figure 10, as-cast, thermally annealed (550 °C, 140 h) and work-hardened Magnox-U samples of identical composition develop markedly different hydride site densities and hydride morphologies after partial reaction under 500 mbar D_2_ at 190 °C, demonstrating that the thermal and mechanical history—rather than the chemical composition alone—exerts first-order control over the incubation period and the spatial distribution of hydride nucleation [62].

The above studies have revealed the regulatory effects of alloying elements on macroscopic corrosion behavior. However, the microscopic mechanisms underlying these effects, particularly the segregation behavior of alloying elements at critical defects such as grain boundaries and how they influence hydrogen accumulation at grain boundaries and the subsequent embrittlement (reduction in interfacial cohesion), require analysis at the atomic scale.

Research has confirmed that the corrosion resistance of metallic uranium can be enhanced through alloying with certain metallic solute atoms. In recent years, based on first-principles methods, researchers have extensively investigated the adsorption and dissociation processes of hydrogen, oxygen, and water molecules on the surfaces of uranium–titanium and uranium–niobium alloys [63,64,65,66,67,68,69,70,71,72]. Liu et al. [63] employed density functional theory to construct two types of uranium–titanium alloy surface models, representing an alloyed surface and a titanium-coated surface, respectively, and examined the effect of titanium layer pretreatment on oxygen adsorption on the uranium surface. They found that titanium layer pretreatment can, to a certain extent, inhibit the diffusion of oxygen atoms on the uranium surface. Cheng et al. [64] employed DFT+U density functional theory to analyze the adsorption characteristics of water on a titanium-doped uranium surface. By investigating the adsorption energies, charge transfer, and electronic structure changes at different adsorption sites, they pointed out that water adsorption on the titanium-doped uranium surface belongs to a mixed mode of physisorption and chemisorption, in which physisorption exhibits a specific equilibrium distance, while chemisorption occurs only at certain sites and predominantly takes the form of dissociative adsorption. Compared with the early studies on the surface corrosion of uranium–titanium and uranium–niobium alloys, investigations into the surface corrosion of uranium–molybdenum alloys induced by small molecules started relatively late. Tian et al. [73] employed first-principles calculations to investigate the adsorption behavior of oxygen on the γ-U(110) surface and the molybdenum-doped γ-U(110) surface. Their results showed that the most stable adsorption site for oxygen atoms on the γ-U(110) surface is the short-bridge site, and molybdenum doping reduces the adsorption energy of oxygen atoms at all adsorption sites. Based on the analysis of oxygen atom migration on the surface, they proposed that molybdenum doping increases the energy barrier for oxygen atom diffusion. The existing theoretical findings provide important microscopic information for understanding the uranium–hydrogen reaction.

### 4.2. Role of Non-Metallic Impurities and Second Phases

During the smelting, processing, and service of metallic uranium, non-metallic elements such as carbon (C), nitrogen (N), and silicon (Si) are inevitably introduced as impurities. The content, existing form, and spatial distribution of these elements exert a significant influence on the hydriding corrosion behavior of the material. Unlike metallic alloying elements, which act primarily through solid solution formation and phase transformations, non-metallic elements regulate the adsorption, diffusion, and reaction pathways of hydrogen mainly by altering the local chemical bonding state, surface reactivity, and defect structure. When carbon is present in the form of amorphous carbon or as a high-concentration solid-solution impurity, it exhibits a strong catalytic effect. On U–0.1 wt.% Cr alloy surfaces, the hydriding corrosion morphology depends markedly on the local carbon impurity content in regions covered by amorphous carbon, as hydrides nucleate and grow rapidly, whereas carbon-poor regions remain much less attacked [74]. This is attributed to the fact that amorphous carbon provides a large number of active sites, which may lower the energy barrier for hydrogen molecule dissociation or modify the electronic work function of the surface, thereby significantly promoting the initial hydriding reaction.

In contrast, when carbon forms stable carbides with uranium or when a carbide layer is formed on the surface through methods such as ion implantation or surface passivation, it significantly impedes the hydriding reaction. For example, the formation of a carbide layer through surface treatment can effectively prolong the incubation period of the hydriding reaction [75]. Carbide phases possess different crystal structures and chemical inertness and may serve as a physicochemical barrier that hinders the permeation of hydrogen into the metallic matrix. Carbon thus exerts two opposing effects—catalyzing hydriding in its amorphous form while inhibiting it in the form of carbides—so that the ultimate corrosion characteristics are determined jointly by the total carbon content and by the state and distribution of carbon in the material.

Silicon, as a common trace impurity, exerts a remarkably pronounced accelerating effect on hydriding corrosion. Studies have confirmed that when the silicon content in uranium increases to approximately 100 ppm, the hydriding reaction rate rises sharply [76]. In-depth theoretical calculations have shown that silicon atoms can significantly reduce the energy barrier for the uranium–hydrogen reaction [77]. The mechanism underlying this catalytic effect may be related to the fact that silicon alters the local electron density or lattice strain, making it easier for hydrogen atoms to combine with uranium atoms to form UH_3_. This effect has been verified in U–Ti alloys under different heat treatment conditions, demonstrating the universality of the influence of silicon [4].

**Figure 11:**
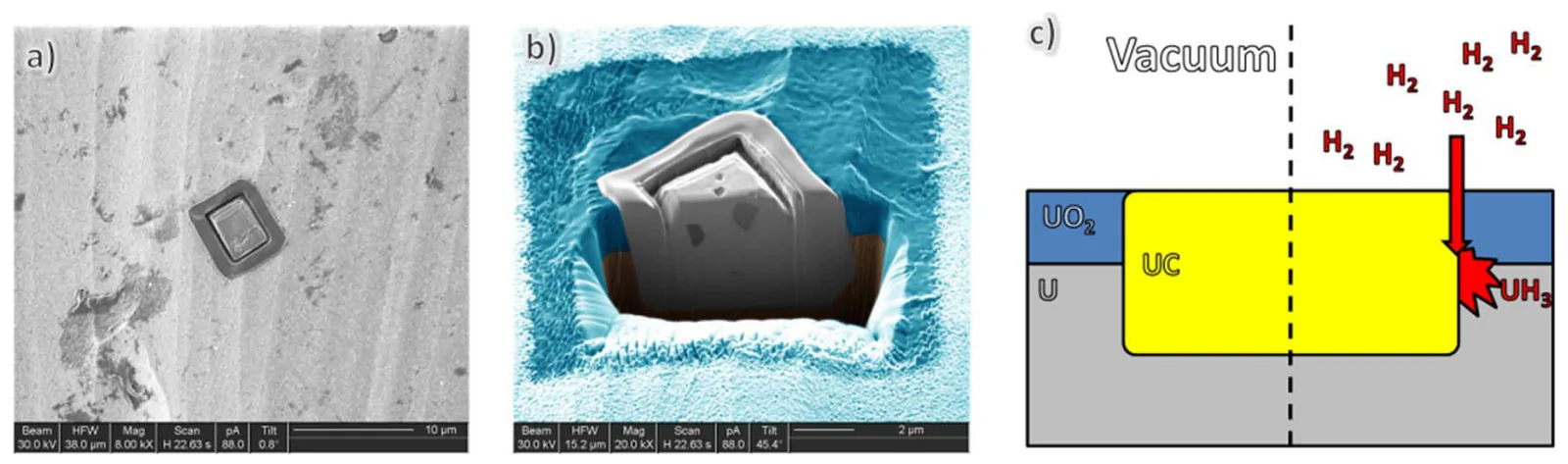
Secondary electron images of an inclusion in uranium metal and a schematic showing how such features offer preferential sites for hydride nucleation. (**a**) A typical inclusion at the uranium surface; (**b**) a cross-section through the same inclusion, showing that it penetrates a locally thick (>1 μm) surface oxide layer (highlighted in blue); (**c**) a schematic showing how the carbide–metal interface allows hydrogen to bypass the oxide layer, making inclusions preferential hydride nucleation sites. Adapted from Banos et al. [78] under the terms of the Creative Commons Attribution 4.0 licence.

Uranium carbide inclusions (e.g., UC, U_2_C_3_) originate from carbon impurities introduced during smelting, casting, and machining—for instance from graphite crucibles, organic contaminants, or carbon-bearing process gases—which react with uranium to form carbide phases. These carbides are often present in the matrix as inclusions. Owing to their differences in thermal expansion coefficient and crystal structure from the uranium matrix, microcracks and stress concentrations readily develop at the inclusion/matrix interface, leading to discontinuity or rupture of the overlying surface oxide film. According to the Glascott model [37], these regions provide fast diffusion pathways for hydrogen atoms, making them preferential sites for hydride nucleation. However, previous studies have shown that not all inclusions are surrounded by corrosion to the same extent; their susceptibility may be further modulated by multiple factors, including the crystallographic orientation of the grain in which the inclusion resides, the specific morphology of the local oxide film, and subtle differences in the distribution of the stress field [25,36]. This mechanism is illustrated directly in Figure 11. Cross-sectional imaging of a typical inclusion shows that it penetrates the surface oxide layer [78], which is locally more than 1 μm thick, so that the carbide–metal interface forms a continuous pathway from the external environment to the metal substrate. Hydrogen can therefore bypass the oxide barrier along this interface and accumulate at depth, which explains why inclusions act as preferential nucleation sites for hydride growth.

Studies have shown that non-metallic impurities such as carbon and silicon can significantly alter the hydriding reaction rate and corrosion morphology of uranium; however, based solely on elemental content and macroscopic kinetics, it is difficult to distinguish their different roles, including surface catalysis, second-phase formation, lattice distortion, and grain boundary segregation. Microscopic characterization reveals that non-metallic impurities in uranium alloys are not uniformly distributed. Kautz et al. [79] employed scanning electron microscopy and atom probe tomography to study as-cast U–10Mo alloy and found that non-metallic inclusions such as C and O are predominantly distributed near grain boundaries, with Si also frequently present as a coexisting impurity, indicating that grain boundaries are important regions for non-metallic element enrichment and the formation of complex local chemical environments.

**Figure 12:**
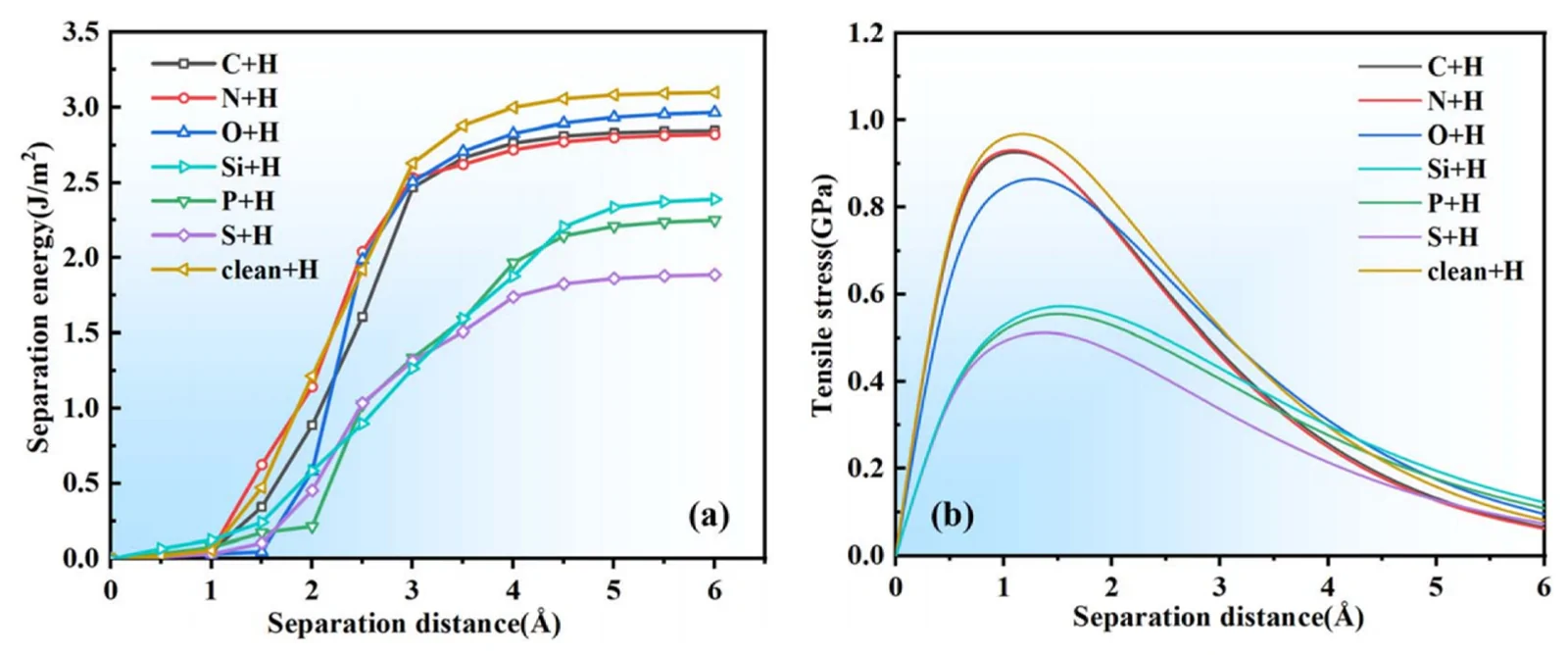
Perfect grain boundaries and non-metallic element-doped grain boundaries: (**a**) separation energy and (**b**) tensile stress plots [16]. In each panel, the curves compare the perfect grain boundary with grain boundaries doped with C, N, O, Si, P, or S, showing that all non-metallic dopants reduce the grain boundary separation energy and cohesive strength.

Recent first-principles studies have further compared the behavior of C, N, O, Si, P, and S at grain boundaries in metallic uranium at the atomic scale [16]. The computational results indicate that all six of the aforementioned elements exhibit a spontaneous tendency to segregate at the studied grain boundaries, and the closer to the grain boundary core, the higher the segregation stability, as shown in Figure 12. This suggests that the influence of non-metallic impurities on hydriding corrosion cannot be understood solely as a bulk solid-solution effect; their role in reconstructing the interfacial structure and electronic states after forming a locally enriched layer at the grain boundary must also be considered. It is worth noting that a reduction in grain boundary energy due to segregation is not equivalent to an improvement in the grain boundary resistance to fracture. Results based on the Rice–Wang model and first-principles tensile tests [16] indicate that all of the non-metallic elements investigated reduce the grain boundary cohesive strength, with the individual-dopant embrittlement potency following Si > S > P > O > N > C and the total co-doped (non-metal + H) potency following P > Si > S > O > N > C. This difference originates primarily from the structural strain arising from solute size mismatch and the distinct effects of different elements on local chemical bonding.

Non-metallic impurities also alter the segregation behavior of hydrogen at grain boundaries. Calculations by Li et al. [16] demonstrated that pre-segregated C, N, and O raise the energetic cost for hydrogen segregation to the grain boundary, exhibiting a certain hydrogen-repelling effect; P and S, in contrast, further enhance hydrogen segregation at the grain boundary, while the influence of Si on hydrogen segregation is relatively weak. However, even if certain elements can suppress further hydrogen enrichment, they may themselves still reduce the grain boundary cohesive strength; therefore, one cannot simply judge whether an element is beneficial for resisting hydrogen embrittlement based on its “hydrogen-repelling” character. More importantly, when hydrogen coexists with non-metallic elements, weak hydrogen–non-metal bonds may replace the originally stronger non-metal–U bonds and reduce the electron density in the grain boundary region, thereby further weakening the grain boundary. This indicates that the regulation of uranium hydriding corrosion by non-metallic elements exhibits a pronounced duality: on the one hand, they modify the thermodynamics of hydrogen segregation, and on the other, they directly modulate the intrinsic cohesive strength of the grain boundary.

The above theoretical results can offer new explanations for existing experimental phenomena. For instance, the acceleration of the uranium hydriding reaction by silicon impurities may be related not only to their potential influence on the local strain required for hydride nucleation, but also to the strong structural mismatch and interfacial weakening they cause at grain boundaries. The role of carbon, on the other hand, depends on its specific existing state: amorphous carbon may promote H_2_ dissociation on the surface, whereas grain-boundary-segregated carbon or carbide inclusions may exert entirely different effects on hydrogen transport and grain boundary strength. Therefore, when evaluating non-metallic impurities, it is necessary to simultaneously distinguish among solid-solution atoms, grain-boundary-segregated atoms, surface carbon layers, and second-phase inclusions; the different chemical states of the same element cannot be reduced to a single promoting or inhibiting effect.

On the whole, simulation studies have advanced the understanding of non-metallic impurities from a macroscopic “content effect” to the microscopic mechanism of “grain boundary segregation–hydrogen co-segregation–interfacial weakening,” but certain limitations remain. Current results are primarily based on a single ideal grain boundary, low solute concentrations, and static zero-temperature models, and have not yet adequately accounted for the effects of different grain boundary types, multi-element competitive segregation, vacancies, or temperature and stress. Future work should systematically compare the segregation of non-metallic elements at general grain boundaries, low-Σ grain boundaries, and twin boundaries, and, in conjunction with hydrogen diffusion barriers and finite-temperature simulations, establish quantitative relationships linking the content and existing forms of non-metallic elements, the grain boundary chemical environment, and the macroscopic hydriding susceptibility. The main effects of the alloying elements and non-metallic impurities discussed above are summarized in Table 1.

### 4.3. Summary

Both metallic elements and non-metallic impurities can significantly modulate the hydriding corrosion of uranium, but their pathways of action differ. Metallic elements such as Nb, Ti, and Mo primarily influence hydrogen adsorption, dissolution, diffusion, and hydride nucleation by altering phase stability, surface reaction barriers, defect structures, and the local electronic environment. Non-metallic impurities such as C and Si, in contrast, exhibit a stronger dependence on their existing state, with their effects involving surface catalysis, grain boundary segregation, second-phase formation, and localized disruption of the oxide film.

Existing research has gradually shifted from merely comparing elemental contents toward analyzing local structures and interfacial chemistry, yet two deficiencies remain. First, most experiments yield correlations between alloy composition, heat-treatment microstructure, and macroscopic hydriding behavior, but it remains difficult to isolate the independent contributions of phase structure, grain boundary segregation, and second-phase interfaces. Second, current calculations have mostly focused on ideal surfaces or single grain boundaries, lacking systematic comparisons among different grain boundary types, multi-element co-segregation, and finite-temperature conditions. Therefore, the design of hydriding-resistant alloys should not merely aim at reducing the overall reaction rate, but should simultaneously consider the effects of elements on grain boundary stability and local hydrogen enrichment. Future work needs to establish a unified evaluation framework linking “elemental segregation tendency, hydrogen segregation regulation, and grain boundary cohesive strength,” and to determine the existing states and effective action sites of elements in combination with the actual microstructure. Elements or element combinations that can strengthen grain boundaries, suppress hydrogen enrichment, and avoid the formation of detrimental second phases represent an important direction for the chemical regulation of grain boundaries in uranium materials.

## 5. Conclusions and Perspectives

In summary, the localized hydriding corrosion of metallic uranium is governed by the coupled behavior of the surface oxide layer and grain boundaries, and can be effectively regulated by controlling the state and distribution of alloying elements and non-metallic impurities. These findings establish grain boundary engineering—through both interfacial structure design and chemical-composition control—as a promising strategy for enhancing the hydriding resistance of uranium materials.

Future research should address three key challenges. First, a comprehensive database of hydrogen segregation and diffusion across different grain boundary types should be established to identify the structural features of the most susceptible boundaries. Second, the continuous transport pathway of hydrogen from the UO_2_ surface, through oxide grain boundaries and the UO_2_/α-U interface, into the metallic grain boundaries remains to be resolved. Third, vacancies, solutes, multiple hydrogen atoms, and stress should be incorporated into a unified model, with robust parameter transfer and cross-validation among first-principles calculations, mesoscopic simulations, and in situ experiments. Addressing these challenges will provide critical support for the safe service and hydriding-resistant design of uranium materials.

## Figures and Tables

**Table 1:** Summary of the effects of alloying elements and non-metallic impurities on the hydriding corrosion of metallic uranium.

Element	Type	Effect on Incubation Period	Effect on GB Cohesion	Mechanism of Action	Ref.
Nb	Metallic alloying	Prolongs	Not reported	Stabilizes γ phase, suppresses γ→α transformation; modifies GB energy states	[61]
Ti	Metallic alloying	Not reported	Not reported	Inhibits O diffusion; alters H_2_O/H_2_ adsorption (physisorption–chemisorption)	[63]
Mo	Metallic alloying	Not reported	Not reported	Reduces O adsorption energy; raises O diffusion barrier	[73]
C (amorphous)	Non-metallic impurity	Shortens	Not reported	Surface catalysis: promotes H_2_ dissociation	[74]
C (carbide, UC/U_2_C_3_)	Non-metallic impurity/second phase	Prolongs (protective layer); shortens (discrete inclusions)	Not applicable (a second-phase inclusion rather than a grain boundary segregant)	Acts as a physicochemical barrier to hydrogen permeation; the carbide/matrix interface develops stress concentration and microcracks that serve as fast hydrogen pathways	[37,75]
Si	Non-metallic impurity	Shortens	Weakens (strong)	Surface catalysis; lowers U–H reaction barrier; strong GB embrittlement	[16,76,77]
N	Non-metallic impurity	Not reported	Weakens (mild)	GB segregation; repels H segregation; size-mismatch strain	[16]
O	Non-metallic impurity	Not reported	Weakens (mild)	GB segregation; repels H segregation	[16]
P	Non-metallic impurity	Not reported	Weakens (strong)	Enhances H segregation; strong GB embrittlement	[16]
S	Non-metallic impurity	Not reported	Weakens (strong)	Enhances H segregation; strong GB embrittlement	[16]

## Data Availability

No new data were created or analyzed in this study. Data sharing is not applicable to this article.
